# Vertical sleeve gastrectomy induces enteroendocrine cell differentiation of intestinal stem cells through bile acid signaling

**DOI:** 10.1172/jci.insight.154302

**Published:** 2022-06-08

**Authors:** Ki-Suk Kim, Bailey C.E. Peck, Yu-Han Hung, Kieran Koch-Laskowski, Landon Wood, Priya H. Dedhia, Jason R. Spence, Randy J. Seeley, Praveen Sethupathy, Darleen A. Sandoval

**Affiliations:** 1 Department of Pediatrics, University of Colorado Anschutz Medical Campus, Aurora, Colorado, USA.; 2 Department of Surgery, University of Michigan, Ann Arbor, Michigan, USA.; 3 Department of Biomedical Sciences, Cornell University, Ithaca, New York, USA.; 4 Department of Surgery, The Ohio State University Comprehensive Cancer Center and The Ohio State University Wexner Medical Center, Columbus, Ohio, USA.; 5 Department of Cell and Developmental Biology, University of Michigan, Ann Arbor, Michigan, USA.

**Keywords:** Cell Biology, Metabolism, Adult stem cells, Peptides, Surgery

## Abstract

Vertical sleeve gastrectomy (VSG) results in an increase in the number of hormone-secreting enteroendocrine cells (EECs) in the intestinal epithelium; however, the mechanism remains unclear. Notably, the beneficial effects of VSG are lost in a mouse model lacking the nuclear bile acid receptor farnesoid X receptor (FXR). FXR is a nuclear transcription factor that has been shown to regulate intestinal stem cell (ISC) function in cancer models. Therefore, we hypothesized that the VSG-induced increase in EECs is due to changes in intestinal differentiation driven by an increase in bile acid signaling through FXR. To test this, we performed VSG in mice that express EGFP in ISC/progenitor cells and performed RNA-Seq on GFP-positive cells sorted from the intestinal epithelia. We also assessed changes in EEC number (marked by glucagon-like peptide-1, GLP-1) in mouse intestinal organoids following treatment with bile acids, an FXR agonist, and an FXR antagonist. RNA-Seq of ISCs revealed that bile acid receptors are expressed in ISCs and that VSG explicitly alters expression of several genes that regulate EEC differentiation. Mouse intestinal organoids treated with bile acids and 2 different FXR agonists increased GLP-1–positive cell numbers, and administration of an FXR antagonist blocked these effects. Taken together, these data indicate that VSG drives ISC fate toward EEC differentiation through bile acid signaling.

## Introduction

Vertical sleeve gastrectomy (VSG) is the most frequently performed bariatric surgery in the United States (1). VSG patients lose approximately 81% of excess body mass within 1 year, and approximately 38% of patients with type 2 diabetes mellitus achieve postoperative remission (2). VSG increases postprandial plasma levels of many gut peptide hormones, such as glucagon-like peptide-1 (GLP-1), gastric inhibitory polypeptide (GIP), peptide YY (PYY), and cholecystokinin (CCK), which all play roles in regulating satiety, lipid handling, energy expenditure, and glucose homeostasis (3). Importantly, this observation is consistent between clinical and preclinical studies, is seen within 2 days following VSG, and is maintained for at least 5 years after surgery (4–8). Therefore, the drastic increase in plasma gut peptides with VSG is presumed to be the mechanism for successful VSG outcomes. However, a critical question that remains is what drives the postoperative increases in circulating gut hormones.

A number of studies (9–11) demonstrated an increase in the number of GLP-1–secreting enteroendocrine cells (EECs) after VSG without any gross morphological changes in the intestinal epithelium. Gut peptides are secreted from EECs, which are scattered individually throughout the intestinal epithelium along with many other intestinal cell types (goblet cells, enterocytes, Paneth cells). The intestinal epithelium is continuously renewed by proliferation and differentiation of crypt base columnar intestinal stem cells (ISCs), which can be isolated and sorted by the marker leucine rich repeat containing G protein–coupled receptor 5 (*LGR5*) (12). The cell signaling pathways that regulate ISC proliferation and differentiation have been well described. First, Notch signaling is required for the renewal of ISCs, and thus the activation of Notch signaling induces cell proliferation (12). In addition, Notch signaling represses the transcriptional regulator atonal bHLH transcription factor 1 (ATOH1) and thereby promotes ISC differentiation toward absorptive enterocytes. Conversely, the inhibition of Notch signaling activates ATOH1 to the differentiation of secretory lineage progenitor cells such as EECs, goblet cells, and Paneth cells (13). Therefore, the *Notch* and *ATOH1* gene families play a significant role in ISC maintenance and EEC differentiation. However, whether VSG drives ISC fate toward EECs, and what signals drive this change, are unknown.

We previously found that a bile acid (BA) receptor, farnesoid X receptor (FXR), is necessary for the VSG-induced weight loss and improvements in glucose homeostasis (14). However, the mechanisms by which FXR mediates the metabolic impact of VSG remain elusive. Like gut peptides, many BA species are increased several-fold after VSG in mice (15) and humans (16). Previous work has demonstrated that the taurine-conjugated BA, taurocholic acid (TCA), stimulates intestinal epithelial cell proliferation by activation of epidermal growth factor receptor (EGFR), while a secondary BA, deoxycholic acid (DCA), inhibits cell proliferation by activating FXR, suggesting that different BA species act via distinct signaling mechanisms to regulate intestinal epithelial homeostasis (17). Moreover, FXR signaling has also been found to regulate ISC proliferation in a colorectal cancer mouse model (18). Thus, accumulating data suggest that BA signaling is critical for VSG outcomes and ISC fate. Hence, we hypothesized that the increased secretion of BAs after VSG drives ISC fate toward EECs through BA signaling, which then results in a drastic increase of various gut peptides.

## Results

**Figure 1:**
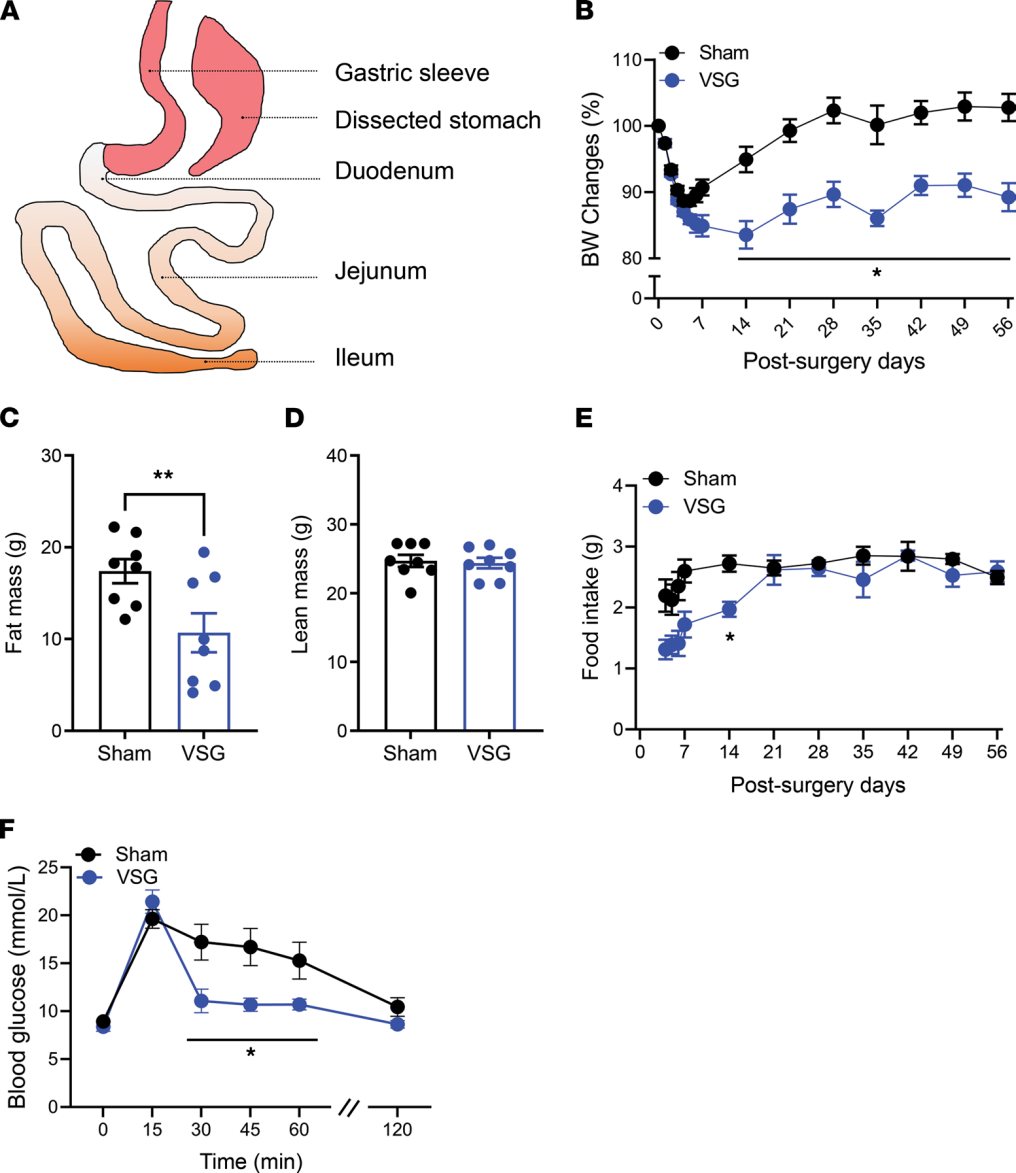
VSG induces metabolic improvements in control male DIO mice. (**A**) A conceptual graphic of VSG procedure. (**B**–**D**) BW change from baseline (**B**), fat mass (**C**), lean mass (**D**), and food intake (**E**) in sham- or VSG-treated mice. (**F**) Oral glucose tolerance (2 g/kg) of sham- or VSG-treated mice. (**B** and **C**) Body composition was measured at 8 weeks after surgery. (**F**) Oral glucose tolerance testing was performed at 5–6 weeks after surgery. Mean ± SEM. Statistics, *t* test or ANOVA. *P < 0.05, **P < 0.01, *n* = 8.

As seen in human bariatric patients (3) and in our previous murine work (19), VSG results in dramatic reductions in body weight (BW) and fat mass without significantly altering lean mass in diet-induced obese (DIO) mice (Figure 1, A–D). VSG-treated mice initially had a significant reduction in food intake, which was restored to the level of their sham-treated counterparts within 3 weeks after surgery (Figure 1E). VSG mice also had improved oral glucose tolerance compared with sham surgery mice (Figure 1F). These data highlight that we have replicated our previous findings regarding the impact of VSG on BW, food intake, and glucose tolerance (19–21).

**Figure 2:**
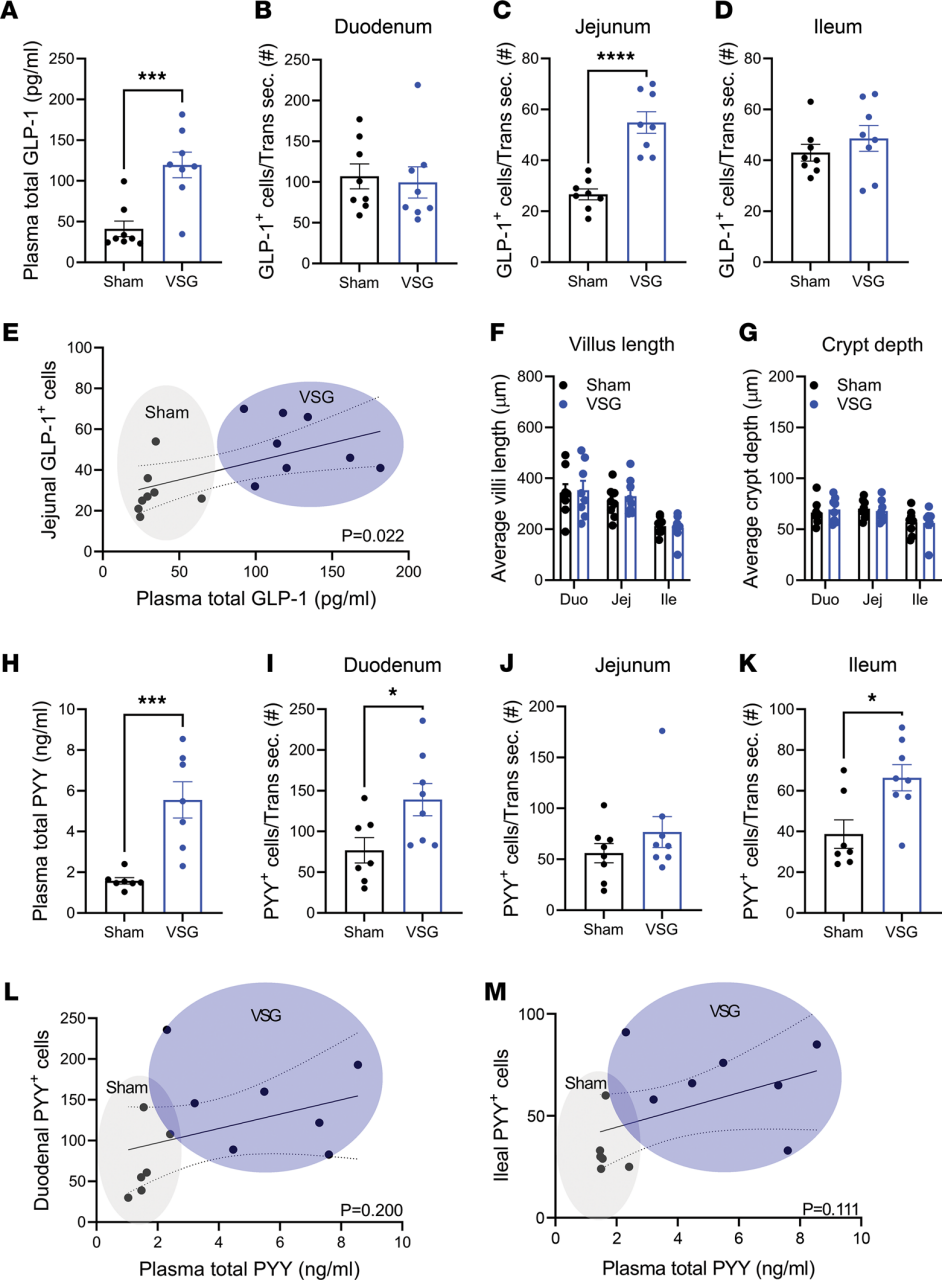
VSG increases plasma levels and EEC number of GLP-1 and PYY. (**A**) Plasma total GLP-1 levels 15 minutes after a mixed liquid meal in sham or VSG-treated mice. (**B**–**D**) GLP-1^+^ EEC number of intestinal transverse sections (Trans sec.) of the duodenum (**B**), jejunum (**C**), or ileum (**D**). (**E**) Linear regression analysis of the jejunal GLP-1^+^ cell number versus plasma total GLP-1 level. (**F** and **G**) The villi length (**F**) and crypt depth (**G**) of the intestinal segments. (**H**) Plasma total PYY levels at 15 minutes after a liquid mixed meal administration in sham or VSG-treated mice. (**I**–**K**) PYY^+^ EEC number of the intestinal transverse sections of the duodenum (**I**), jejunum (**J**), or ileum (**K**). (**L** and **M**) Linear regression analysis of the duodenal (**L**) or ileal (**M**) PYY^+^ cell number versus plasma total PYY level. Mean ± SEM. Statistics, *t* test or ANOVA. *P < 0.05, ****P < 0.0001, *n* = 8.

Here, we focus on Glp-1 and Pyy, as these 2 anorectic gut peptides are historically suggested to be secreted from the same cells, have demonstrated effects on suppressing feeding, and are increased postprandially by VSG. Consistent with our and others’ previous studies (19), plasma total Glp-1 levels were increased 15 minutes after oral administration of a mixed liquid meal (Ensure plus) in VSG versus sham mice (Figure 2A). We also observed that Glp-1^+^ cell numbers were 2-fold higher in VSG versus sham mice, though this was observed specifically within the jejunal epithelium, with no significant increase in the duodenal or ileal epithelia (Figure 2, B–D). The number of jejunal Glp-1^+^ EECs was significantly associated with plasma levels of total Glp-1 (Figure 2E). Notably, we did not observe any changes in villi length or crypt depth in any part of the small intestinal sections (Figure 2, F and G), indicating that the increased Glp-1^+^ EEC number was not the result of intestinal proliferation. Plasma levels of Pyy were also increased in VSG-treated mice (Figure 2H). Intriguingly, we observed Pyy^+^ EEC number was significantly increased in the VSG-treated duodenal and ileal epithelia, but not in the jejunal epithelium (Figure 2, I–K), demonstrating a regional difference in VSG-induced changes within subtypes of EECs. The number of duodenal or ileal Pyy^+^ EECs did not significantly correlate with the plasma levels of total Pyy (Figure 2, L and M), even when the duodenal and ileal cell numbers were pooled (*P* = 0.08). These data suggest that intestinal cellular adaptations to VSG drive increases in postprandial GLP-1 levels.

**Figure 3:**
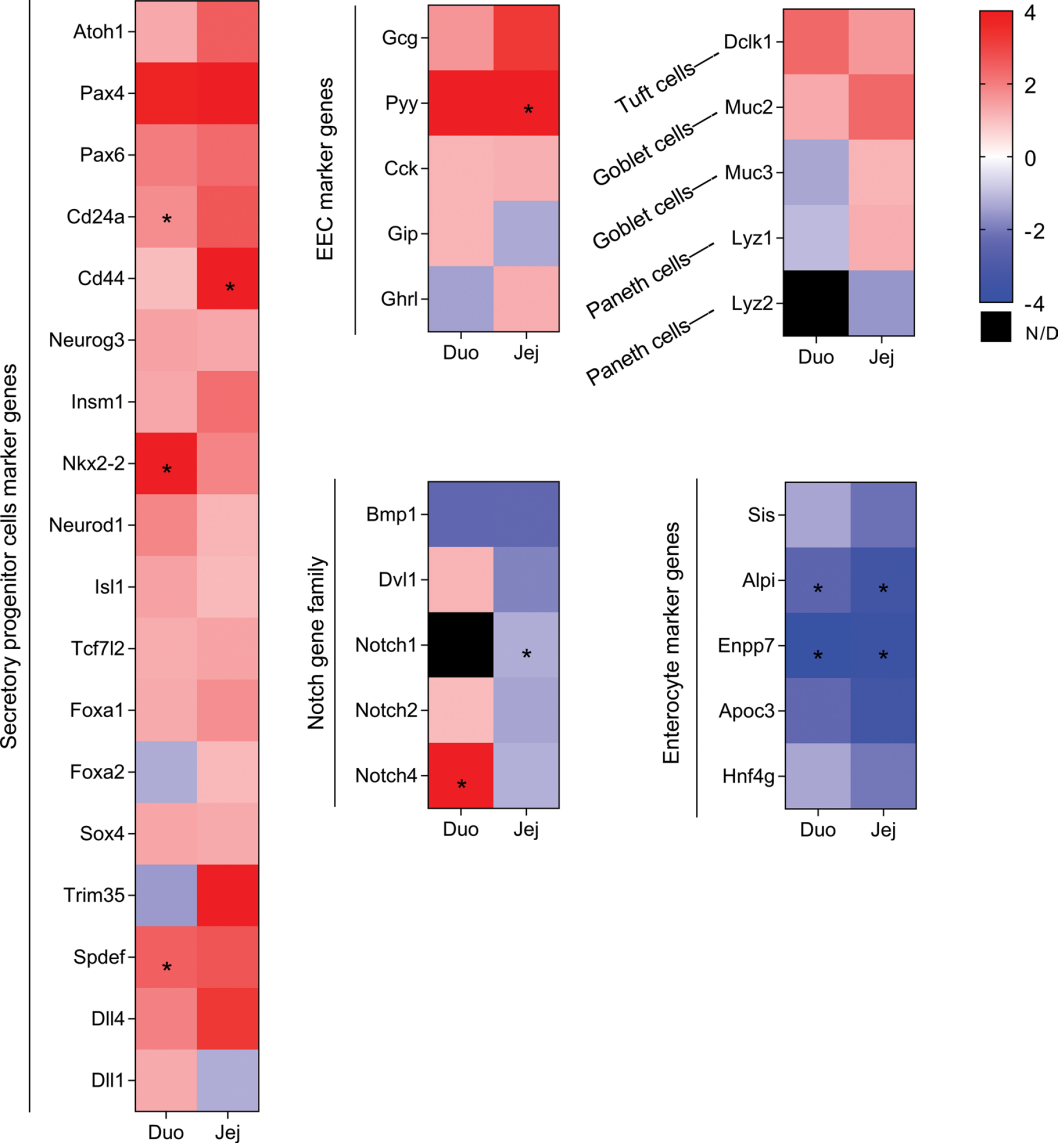
Heatmap analysis of the RNA-Seq results of the sham or VSG-treated Lgr5-EGFP^+^ cells in duodenum and jejunum. Color denotes variation across stages (normalized by DESeq2). Sham (*n* = 2) and VSG (*n* = 4) were used for RNA-Seq. Statistical significance in VSG versus sham is denoted as an asterisk when adjusted *P* value is less than 0.2. N/D, not detected.

Given the high turnover rate of cells within the intestinal epithelium, cellular proliferation and differentiation is a highly regulated process. To determine whether the increase in EECs after VSG is due to alterations in differentiation, we performed VSG on a genetically modified mouse model expressing EGFP in its *Lgr5* gene (Supplemental Figure 1, A and B; supplemental material available online with this article; https://doi.org/10.1172/jci.insight.154302DS1), a specific ISC/progenitor marker gene (12). As expected (19–21), the VSG-treated Lgr5^EGFP^ mice demonstrated reductions in BW and fat mass, normal lean mass, and improvements in oral glucose tolerance compared with their sham counterparts (Supplemental Figure 1, C–F). We then performed RNA-Seq on the GFP^+^ ISCs sorted from the duodenal, jejunal, and ileal epithelia. We presorted immune cells (CD45 positive), endothelial cells (CD31 positive), apoptotic (annexin V positive), and dead cells (stained with Sytox blue) (Supplemental Figure 2A) and saw no effect of surgery on these cell populations. We did observe that the proportion of GFP^+^ ISCs was decreased in the duodenum and jejunum, but not ileum, in VSG-treated mice (Supplemental Figure 2B). Principal component analysis of the transcriptomic profiles stratified sham- and VSG-treated mice across all segments (Supplemental Figure 3, A–C), demonstrating the differential impact of VSG on the various sections of the small intestine. By performing differential expression analysis (*P* < 0.05, adjusted *P* < 0.2, log_2_ fold change > 0.5, normalized count > 50 by DESeq2), we found that VSG altered 467 genes (259 up; 208 down) in the duodenum, 510 genes (348 up; 162 down) in the jejunum, and 955 genes (600 up; 355 down) in the ileum (Supplemental Figure 3, D–F). Of note, some of the VSG-increased genes were region specific (Supplemental Figure 3, G and H). Consistent with our observation that VSG promoted allocation of EECs in the small intestine, we found that GFP^+^ ISCs, particularly from duodenal and jejunal regions, exhibited elevated expression patterns of genes that mark and/or regulate secretory lineage differentiation after VSG (Figure 3). These include genes that encode proteins involved in secretory progenitor cell fate control (*Atoh1*, ref. 12; and its target genes *Spdef*, *Dll3*, and *Dll4*) and EEC differentiation (*Pax4*, ref. 22; and *Pax6*, ref. 23), as well as gut peptides (*Gcg*, *Pyy*, *Cck*, *Gip*, and *Fgf15*). In contrast, enterocyte marker genes (*Sis*, *Alpi*, *Enpp7*, and *Apoc3*) were decreased in GFP^+^ ISCs of duodenal and jejunal samples from VSG mice (Figure 3). Of note, several genes involved in Notch signaling, a pathway critical for priming ISCs toward the absorptive lineage (13), were remarkably decreased after VSG, especially in the jejunum (Figure 3).

We validated the RNA-Seq data with quantitative PCR and confirmed that *Gcg* (GLP-1–encoding gene), but not *Pyy*, was significantly increased in the jejunal ISCs of VSG-treated versus sham-treated mice (Supplemental Figure 4). Though we observed an upregulation of the classic marker of goblet cells (*Muc2*) across all 3 regional segments (Figure 3), this change did not lead to the corresponding increase in the protein expression (Supplemental Figure 5). These data suggest that VSG induces ISC fate toward EEC differentiation and that there are critical regional differences among ISCs in the duodenum, jejunum, and ileum in the regulation of Glp-1^+^ and Pyy^+^ cell differentiation.

**Figure 4:**
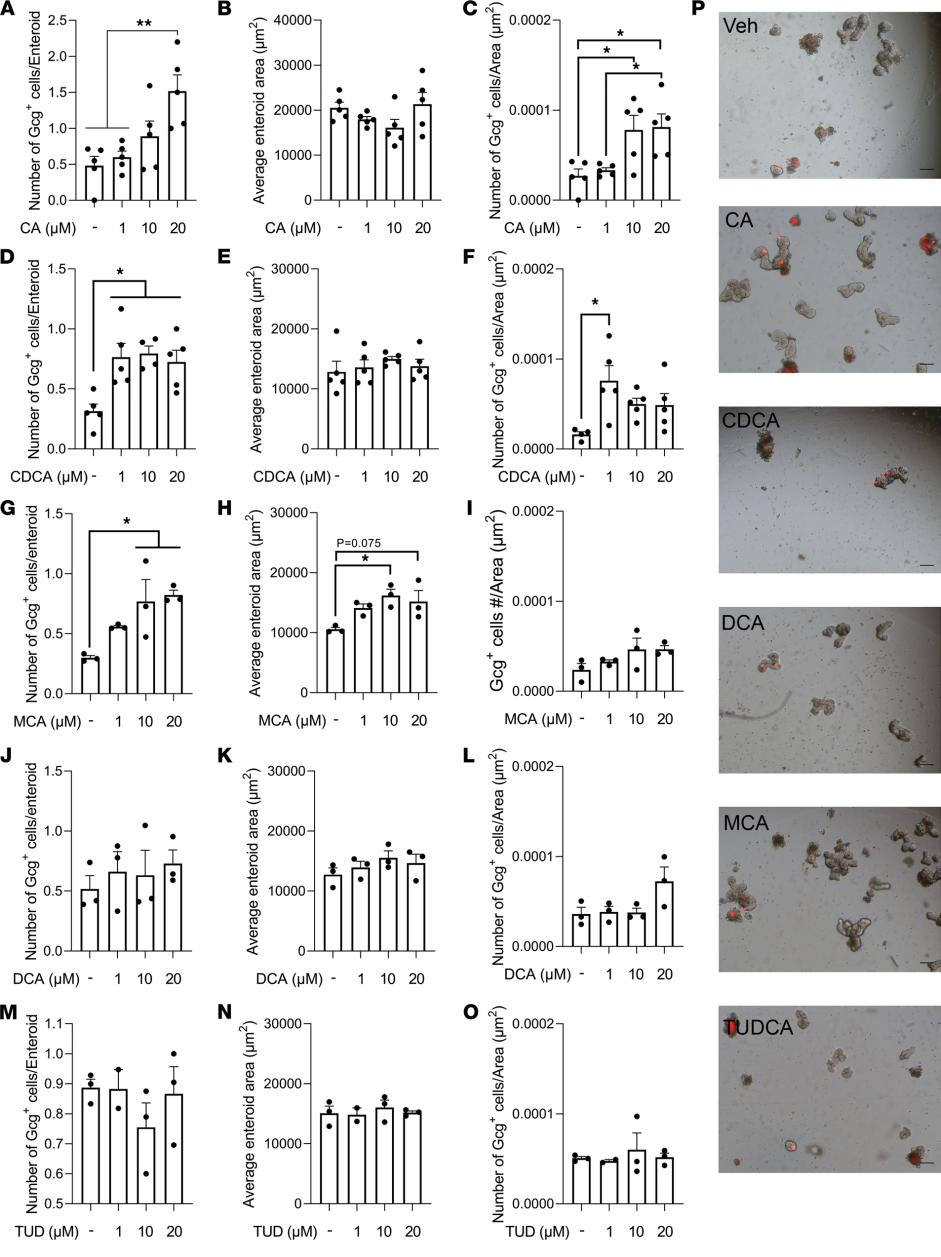
Specific BA treatment increased the growth and GLP-1^+^ EEC number in the Gcg^Tomato^ (GcgCreERT2 crossed with Rosa26 tdTomato) mouse–derived enteroids. Mouse duodenal enteroids were treated with cholic acid (CA; **A**–**C**), chenodeoxycholic acid (CDCA; **D**–**F**), muricholic acid (MCA; **G**–**I**), deoxycholic acid (DCA; **J**–**L**), or tauroursodeoxycholic acid (TUD; **M**–**O**) for 24 hours. The average enteroid area (**A**, **D**, **G**, **J**, and **M**), GLP-1^+^ cell number per enteroid (**B**, **E**, **H**, **K**, and **N**), and GLP-1^+^ cell number per enteroid area (GLP-1^+^ cell density; **C**, **F**, **I**, **L**, and **O**) were measured. *n* = 3–5 wells/group; each well contains 20 enteroids. Mean ± SEM. Statistics, ANOVA. *P < 0.05, **P < 0.01. (**P**) Representative images of enteroids treated with vehicle (Veh), CA, CDCA, DCA, MCA, or TUDCA (all 20 μM) for 24 hours. Scale bar: 100 μm.

BAs are found in both intestinal chyme and serum and are significantly increased after VSG (3). Therefore, we treated mouse duodenal derived enteroids with various primary BAs, including cholic acid (CA), chenodeoxycholic acid (CDCA), and muricholic acid (MCA); a secondary BA, DCA; and a tertiary BA, tauroursodeoxycholic acid (TUDCA). We examined enteroid growth and changes in GLP-1^+^ cell number. We observed that CA dose-dependently increased GLP-1^+^ EEC number and density, while it did not affect duodenal enteroid area (Figure 4, A–C). Additionally, CA treatment dose-dependently increased the proportion of the enteroids containing GLP-1^+^ EECs (Supplemental Figure 6A). CA treatment had similar effects when administered to mouse jejunum-derived enteroids (Supplemental Figure 6B). CA treatment also increased expression of genes involved in EEC differentiation in duodenum- and jejunum-derived enteroids (Table 1). Interestingly, a potent GLP-1 receptor antagonist, exendin 9-39 (Ex9), blocked the effect of CA treatment in increasing GLP-1^+^ EECs’ number and density (Supplemental Figure 7), suggesting that GLP-1 receptor signaling is involved in BA-induced EEC differentiation. Another primary BA, CDCA, increased GLP-1^+^ EEC number and density at a lower dose (1 μM) compared with CA (20 μM), while it did not affect the enteroid area (Figure 4, D–F). However, CDCA treatment did not show the same dose dependency, nor did it show greater efficacy than the CA treatment (approximately 2-fold increase with CDCA vs. 3-fold with CA). MCA, another major primary BA in mice, dose-dependently increased GLP-1^+^ EEC number and enteroid growth simultaneously, and thus it did not change GLP-1^+^ EEC density (Figure 4, G–I, and Supplemental Figure 8). DCA and TUDCA, a secondary and tertiary BA, respectively, did not affect GLP-1^+^ number or density, nor did these BAs affect enteroid area (Figure 4, J–O). These data suggest that different BA species have differential roles in regulating ISC fate and that CA is particularly potent at driving increases in GLP-1^+^ cells. See Figure 4P for representative images of enteroids after each BA treatment.

**Figure 5:**
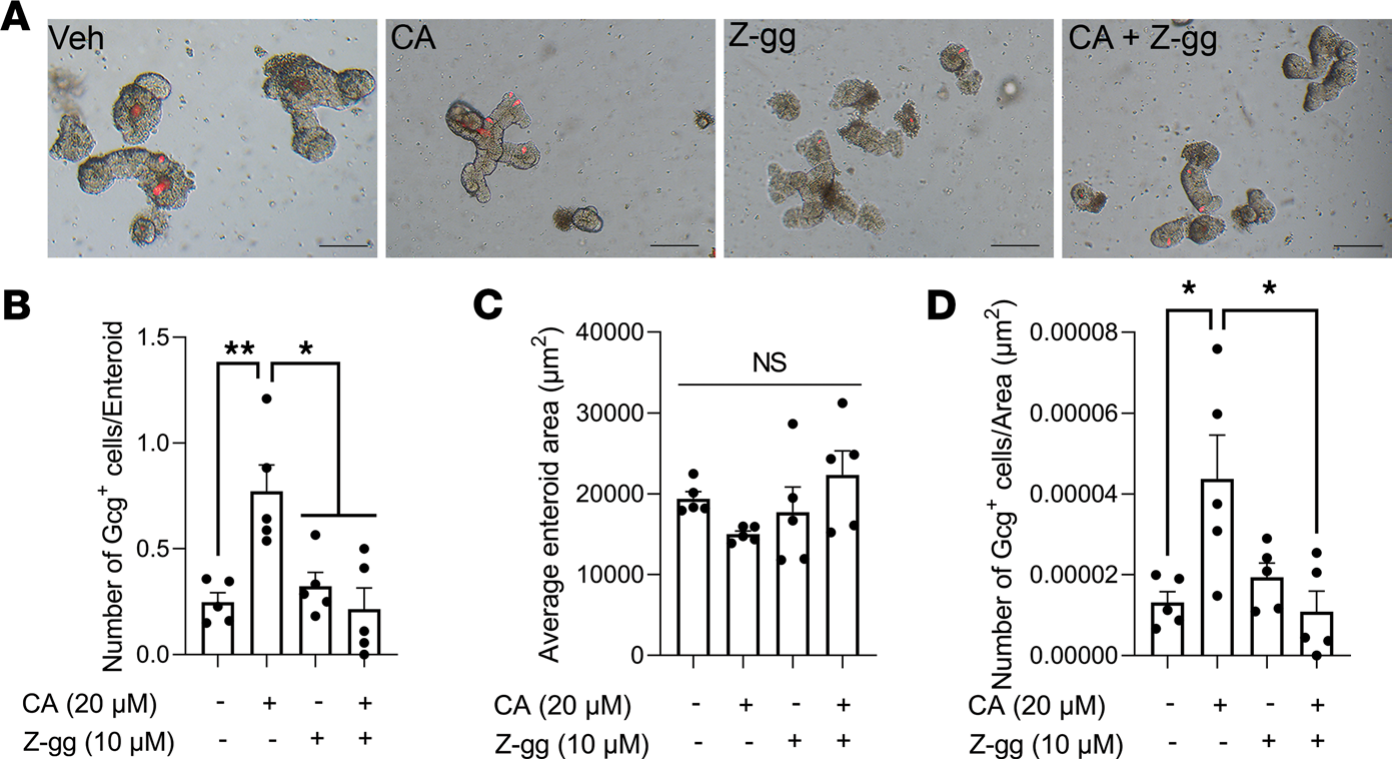
CA treatment increases GLP-1^+^ EEC number in an FXR-dependent manner. (**A**–**D**) CA or a potent FXR antagonist, Z-guggulsterone (Z-gg), was administered to Gcg^Tomato^ mouse–derived enteroids for 24 hours, and the average enteroid area (**B**), GLP-1^+^ cell number per enteroid (**C**), and GLP-1^+^ cell number per enteroid area (GLP-1^+^ cell density; **D**) were measured. Scale bar: 100 μm. *n* = 5 wells/group; each well contains 20 enteroids. Mean ± SEM. Statistics, ANOVA. *P < 0.05, **P < 0.01.

**Figure 6:**
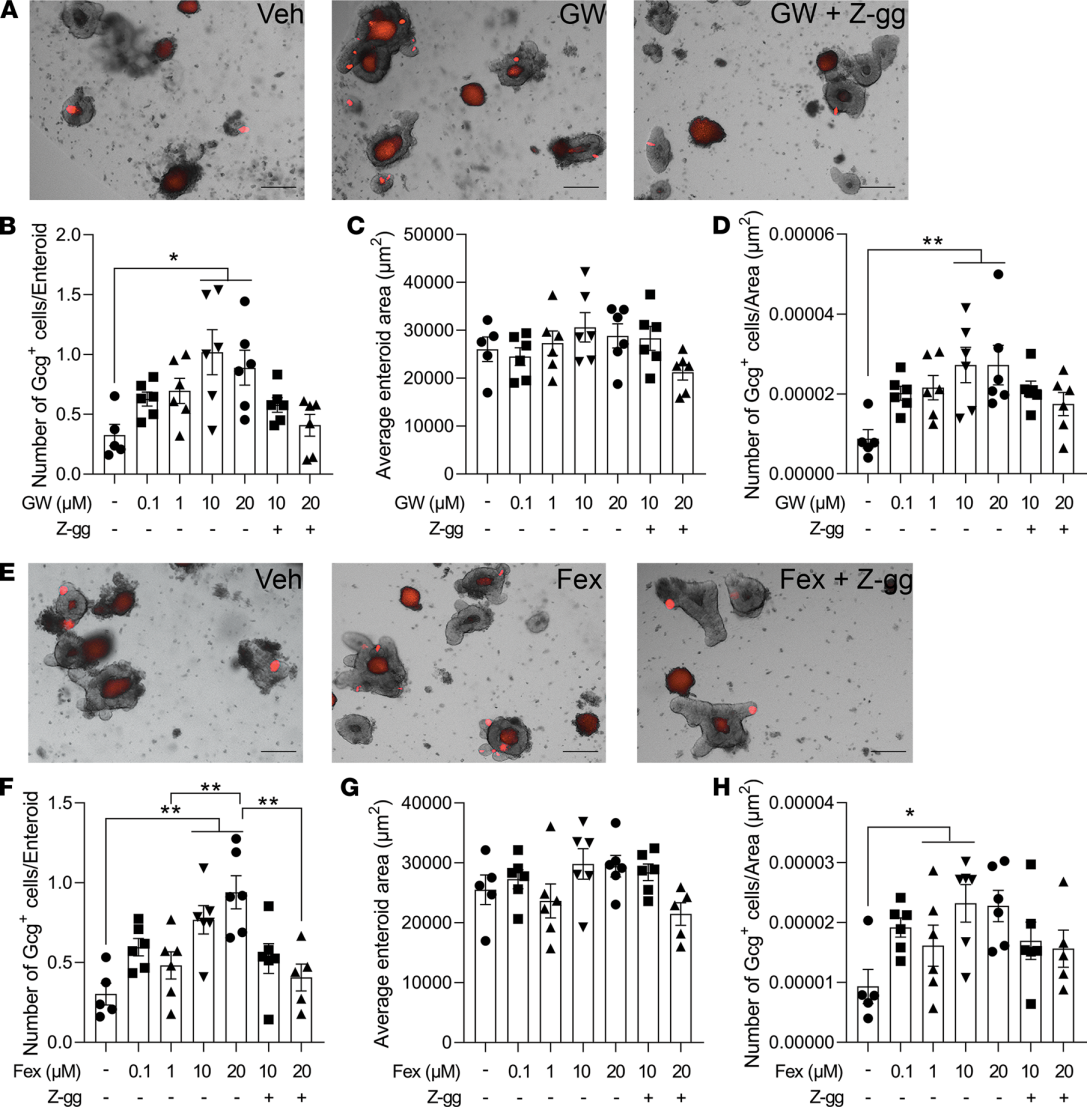
FXR agonist treatment increases GLP-1^+^ EEC number and density. (**A**–**H**) Two potent FXR agonists, GW4064 (GW; **A**–**D**), or fexaramine (Fex; **E**–**H**) alone or in combination with Z-guggulsterone (Z-gg; 10 μM), were administered to Gcg^Tomato^ mouse–derived enteroids for 24 hours. (**A** and **E**) Representative images of enteroids treated with vehicle (Veh), either GW (**A**, 20 μM) or Fex (**E**, 20 μM) alone, or either GW (**A**, 20 μM) or Fex (**E**, 20 μM) in combination with Z-gg for 24 hours. The average enteroid area (**B** and **F**), GLP-1^+^ cell number per enteroid (**C** and **G**), and GLP-1^+^ cell number per enteroid area (GLP-1^+^ cell density; **D** and **H**) were measured. Scale bar: 100 μm. *n* = 6 wells/group, each well contains 20 enteroids. Mean ± SEM. Statistics, ANOVA. *P < 0.05, **P < 0.01.

Notably, we found that a number of BA receptor mRNA transcripts were highly expressed in the *Lgr5*^+^ ISC/progenitor cells throughout the small intestine (Table 2). These BA receptor genes include *Fxr*, epithelial growth factor receptor (*Egfr*), pregnane X receptor (*Pxr*), liver X receptor (*Lxr*), vitamin D receptor (*Vdr*), constitutive androstane receptor (*Car*), and retinoid X receptor (*Rxr*) (Table 2), indicating that BAs could regulate ISC fate via a variety of receptors. We also found mRNA transcripts of other nuclear receptors that have been suggested to be related to FXR, such as the glucocorticoid (*Nr3c1*) and mineralocorticoid receptors (*Nr3c2*), whereas we did not detect expression of the progesterone receptor (*Pgr*), androgen receptor (*Ar*), or estrogen receptor-α (*Esr1*) (Table 2). Because our previous work demonstrated that FXR is necessary for metabolic improvements with VSG, we next tested whether BAs increase GLP-1^+^ EEC number in an FXR-dependent manner. To do this, we administered CA with an FXR antagonist, Z-guggulsterone (17, 24), to mouse-derived enteroids. Administration of Z-guggulsterone alone did not independently influence enteroid area or EEC number (Figure 5, A–D). This is important as Z-guggulsterone has known effects on other steroid receptors, including glucocorticoid, mineralocorticoid, progesterone, androgen, and estrogen receptors. Instead, Z-guggulsterone only had an effect when administered with CA, and in that case it completely blocked the ability of CA to increase GLP-1^+^ EEC number and density (Figure 5, A–D). Because previous data suggest that Z-guggulsterone activates rather than antagonizes PXR (25–27) and CA also activates PXR (28), we predict that CA receptor activation through FXR is necessary for BA-stimulated increases in GLP-1^+^ EEC differentiation. We also observed that 2 potent Fxr agonists, GW4064 (27) and fexaramine (29), increased GLP-1^+^ EEC number and density in a dose-dependent manner, and this effect was blocked by Z-guggulsterone (Figure 6). These data indicate that activation of Fxr can lead to the increase in GLP-1^+^ EEC number and density.

## Discussion

The dramatic increase in postprandial gut peptide levels is observed after rodent VSG models and in patients who undergo bariatric surgery (3). These gut peptides have roles in regulating various aspects of energy homeostasis and blood glucose (30). Despite the fact that the beneficial effects of VSG depend on FXR (14), the target organ for this BA signaling effect remains elusive. Fundamental questions remain regarding what signal drives the increase in gut peptide levels after VSG and whether this phenomenon is linked to BA-FXR signaling. Here, we demonstrate that VSG increases GLP-1^+^ and PYY^+^ EEC numbers and shifts the gene expression profiles of Lgr5^+^ ISC/progenitor cells toward EEC differentiation. We also demonstrate in enteroids that CA increases GLP-1^+^ EECs, that this is blocked by the FXR antagonist Z-guggulsterone, and last that activation of FXR also increases in GLP-1^+^ EECs. Thus, we predict that FXR activation is necessary for VSG-induced increases in GLP-1^+^ cells.

Our group previously demonstrated that gastric emptying rate (GER) is increased after VSG (31). Many have hypothesized that increases in GER raise gut hormone levels through the early exposure of the proximal (where the majority of GIP and CCK-secreting cells are located) and distal intestinal epithelium (where it is believed the majority of GLP-1– and PYY-secreting cells are located) (32) to ingested nutrients. However, we found that the same dose and rate of infusion of dextrose directly into the duodenum still cause significantly greater GLP-1 levels in VSG versus sham animals (31). These data suggest intestinal adaptations drive the increase in postprandial gut peptide secretion. Our current data suggest that the increased drive toward differentiation of EECs is one such adaptation.

Our murine VSG data demonstrate an increased number of EECs, consistent with some previous studies in rodents (9–11) and patients (33). However, one study did not observe this increase in EEC number in a rat model of VSG (34). The cause of this discrepancy is unclear. While other rodent VSG studies maintained animals on the same presurgical high-fat diet (HFD) after surgery, Mumphrey et al. gave their animals a choice between regular chow and HFD postoperatively (34). HFD and long-chain fatty acids stimulate ISC proliferation (35) and obesity (33). Because VSG (36) (and another bariatric surgery, Roux en Y gastric bypass; ref. 37) shifts food preference away from fat-rich foods, the reduction in HFD consumption could independently affect ISC fate. Surprisingly, the effect of obesity on ISC fate is reverted by VSG in humans (33). These data suggest that diet and surgery regulate the composition of circulating factors, which, in turn, regulates ISC fate.

Specific BA subtypes are increased several-fold in sera and jejunum of VSG-treated mice (15) and in patients who undergo VSG (16). A growing body of evidence reveals that BAs are not only lipid emulsifiers but also key signaling molecules with multiple target organs. In particular, studies have demonstrated that BAs regulate ISC fate through FXR and EGFR. An FXR agonist (18) or specific BAs that activate FXR (e.g., TCA) (17) inhibit intestinal epithelial proliferation, whereas other BAs that inhibit FXR signaling (MCA and DCA) increase intestinal proliferation (18), likely through an EGFR-dependent manner (17). None of these studies evaluated the role of BA receptors on specifically regulating EEC differentiation. Here, we demonstrated that a primary BA, CA, as well as 2 different Fxr agonists, increased GLP-1^+^ EECs, and Z-guggulsterone blocked this effect. Altogether, our data suggest that FXR activation activates EEC differentiation pathways while inhibiting ISC proliferation.

A recent study has demonstrated that the BA subtype lithocholic acid and a GPBAR1 (a.k.a. TGR5) agonist increased GLP-1^+^ cell numbers in mouse-derived enteroids in a GLP-1 receptor–dependent and a serotonin 5-hydroxytryptamine receptor 4–dependent (5-HT4–dependent) manner (38). Conversely, our study demonstrated that the CA-induced increase in GLP-1^+^ cells was blocked by antagonizing the GLP-1 receptor; however, we did not find transcripts for GPBAR1 or GLP-1 receptor within ISCs (see Table 2). Therefore, it is possible that other intestinal epithelial cells that express GLP-1 receptor indirectly regulate EEC differentiation of ISCs. The 5-HT4 gene (*Htr4*) is expressed in ISCs in the duodenum and jejunum (though not in ileal ISCs), but the numbers of the transcripts were very low (152 and 29 transcripts, respectively). Still, it is possible that some BAs directly target ISCs to increase GLP-1^+^ cell number through the 5-HT4 receptor, or they indirectly affect ISC fate through the enterochromaffin cells (that secrete serotonin).

In this study, we demonstrated that VSG changes ISC fate toward EEC differentiation. This change of ISC fate accompanies the upregulation of genes involved in EEC differentiation, the downregulation of genes within the Notch family (the signaling pathway that regulates ISC proliferation and enterocyte differentiation) (13, 39), and the downregulation of enterocyte marker genes. However, there are many different types of EECs, and interestingly, we saw a regional difference between changes in the number of GLP-1^+^ and PYY^+^ cells. The reason for this is unclear. Our RNA-Seq analysis reveals that *Foxa2* (a late EEC differentiation marker, ref. 40) and *Trim35* (an L cell marker, ref. 40) were downregulated, and *Dll1* (an early EEC differentiation marker, ref. 40; as well as a Notch ligand, ref. 39) was upregulated within the duodenal versus jejunal ISCs. Also, the expression of Notch family genes, *Notch1*, *Notch2*, and *Notch4*, was downregulated by VSG in the jejunum but not the duodenum. Whether these genes are responsible for the differentially expressed EECs across differing regions of the intestine requires further investigation.

Despite the fact that CA has a similar efficacy in increasing GLP-1^+^ cell number in both duodenal and jejunal derived enteroids, we only observed an increase in GLP-1^+^ cell number in the jejunum of VSG-treated mice. Previous work has shown that the levels of unconjugated BAs, especially of primary BAs (CA and MCA), drastically increase in the jejunum of VSG-treated mice compared with pair-fed, sham-treated mice, and this phenomenon is not observed in the ileum (unclear about the duodenum) (41). Therefore, it is possible that the difference in BA levels between intestinal regions contributed to the difference in GLP-1^+^ cell number. Why specific BA levels are increased in distinct regions of the intestine after VSG, and whether this change is related to the level of EEC differentiation, require further investigation.

Furthermore, the increase in jejunal GLP-1^+^ cell number after VSG might have clinical implications. A recent study revealed that jejunal GLP-1^+^ cell number is decreased in obese patients (even more so in obese patients with type 2 diabetes mellitus, T2DM) compared with nonobese individuals (42). Jejunal EECs isolated from patients with T2DM also demonstrate downregulation in genes involved in EEC differentiation (including *PAX4* and *FOXA2*) and in the *GCG* gene itself (42), indicating that jejunal GLP-1^+^ cell differentiation and number are negatively affected by both obesity and T2DM.

Many studies, including our previous studies, revealed that GLP-1 or PYY, in and of themselves, are not necessary for the metabolic effects of VSG (19, 43, 44). However, many genetic mouse models that target one gut peptide have a compensatory increase in the secretion of other gut peptides (e.g., Gcg-KO mice have a compensatory increase in PYY and GIP levels; ref. 19). Meanwhile, exogenous administration of a combination of multiple gut peptides (GLP-1, PYY, and oxyntomodulin) (45) or combined agonists that target multiple gut peptides (46) has synergistic effects on weight loss and improvements in glycemia. Thus, it may be that surgery-induced increases in multiple gut peptides are necessary for the complete metabolic success of VSG. In addition, the multitargeting effects of bariatric surgery may play a role in orchestrating the metabolic improvements after VSG.

Besides its potent action in antagonizing BA receptor FXR (24), Z-guggulsterone is known to interact with other nuclear receptors. Z-guggulsterone antagonizes glucocorticoid, mineralocorticoid, and androgen receptors (26, 27), while it activates progesterone receptor, estrogen receptor-α, and another BA receptor, PXR (25–27). While we find that the glucocorticoid receptor, mineralocorticoid receptor, and *Pxr* are expressed in ISC, we found negligible expression of the androgen receptor, progesterone receptor, or estrogen receptor-α. Further, we did not observe any significant changes in EEC number or growth in enteroids treated with Z-guggulsterone alone, suggesting the results we see are based on its ability to antagonize CA signaling. As both Z-guggulsterone (25–27) and CA (28) activate rather than inhibit PXR, we predict that the ability of Z-guggulsterone to block the effect of CA is via its actions on FXR. Last, although we cannot exclude the possibility that specific BAs affect EEC differentiation through glucocorticoid and/or mineralocorticoid receptor activation, we are not aware of any data suggesting that BAs signal directly through these receptors.

In conclusion, VSG increases postprandial plasma gut peptide levels, and our data indicate that this is the result of increased number of EECs. Furthermore, we demonstrated that the increase in EEC number is driven by VSG-induced changes in ISC fate toward EEC differentiation in an FXR-dependent manner.

## Methods

### Reagents.

CA (catalog C1129), CDCA (catalog C9377), MCA (catalog SML2372), DCA (D2510), TUDCA (catalog 580549), 4-hydroxytamoxifen (catalog 5082250001), fexaramine (catalog SML1390), GW4064 (catalog G5172), and Z-guggulsterone (catalog 370690) were purchased from MilliporeSigma. Ex9 was purchased from Bachem (catalog 4017799).

### Antibodies.

The following antibodies were used in this study: rabbit anti–GLP-1 (Peninsula Laboratories, T-4363); rabbit anti-PYY (Abcam, ab22663); rabbit anti-Muc2 (Abcam, ab97386); rat anti-CD45–APC (BioLegend, 103111); rat anti-CD31–APC (BioLegend, 102409); APC Annexin V apoptosis detection kit with 7-AAD (BioLegend, 640930); Sytox Blue Dead Cell Stain for flow cytometry (Thermo Fisher Scientific, S34857); donkey anti-rabbit conjugated with Alexa Fluor 488 (Thermo Fisher Scientific, A21206); and goat anti-rabbit IgG conjugated with Cy3 (Thermo Fisher Scientific, A10520).

### TaqMan probes.

Reverse transcription of mRNA was performed with the high-capacity RNA to cDNA kit (Life Technologies). TaqMan probes for *Gcg* (Mm01269055_m1) and *Pyy* (Mm00520716_g1) genes and TaqMan Gene Expression Master Mix (Life Technologies) were used per the manufacturer’s protocol on a Bio-Rad CFX96 Touch Real Time PCR Detection System (Bio-Rad Laboratories). Reactions were performed in triplicate using *B2M* as the normalizer.

### Animals.

All animals were single-housed under a 12-hour light/12-hour dark cycle with ad libitum access to water and standard chow (Envigo Teklad; catalog 7012) or 60% HFD (Research Diets; catalog D12492). The animal room was maintained at 25°C with 50%–60% humidity. All studies were performed using animals 8–16 weeks of age, and all animals were euthanized with CO_2_ inhalation.

Male control mice (VilCre; ref. 19) were maintained on 60% HFD for 6 weeks to induce obesity, were matched for BW and body fat, and then received sham or VSG surgery as described previously (19). Briefly, a small laparotomy incision was made in the abdominal wall of the anesthetized mice, and the lateral 80% of the stomach along the greater curvature was excised using an ETS 35 mm staple gun (Ethicon Endo-Surgery). The sham surgery involved the application of gentle pressure on the stomach with blunt forceps. During the first 3 days of the postoperative period, the animals were fed a liquid diet (Osmolite 1.0 Cal, Abbott Nutrition) and then were returned to the 60% HFD. BW was monitored for 8 weeks after surgery. Body composition was measured using an EchoMRI (Echo Medical Systems) before and 8 weeks postoperatively. Five to 6 weeks after surgery, we performed an oral glucose tolerance test (OGTT) following a 5- to 6-hour fast (2 g/kg of 50% dextrose). Female Lgr5^EGFP^ mice (47) were maintained on 60% HFD for 16 weeks to induce obesity, were matched for BW, and then received sham or VSG surgery as previously described. BW was monitored for 4 weeks after surgery, and the body composition was measured before and 4 weeks after surgery. An OGTT was performed 4 weeks after surgery. Male or female C57BL/6J (The Jackson Laboratory) and Gcg^Tomato^ (GcgCreERT2 mice, ref. 19; crossed with Rosa26-tdTomato mice, The Jackson Laboratory) were utilized to generate mouse enteroids (see below for enteroid methods).

### Gut peptide assay.

At 8 weeks after surgery, control mice were fasted for 5–6 hours and were orally administered a liquid mixed meal (200 μL Ensure plus supplemented with 30 mg dextrose). At 15 minutes later, mice were euthanized with CO_2_ gas. The chest cavity was immediately opened, and blood was collected from the left ventricle by cardiac puncture using EDTA-coated tubes containing DPP-4 inhibitor and aprotinin. Total GLP-1 (MesoScale Discovery, catalog K150JVC) was assayed using a sandwich ELISA kit, whereas total PYY (Crystal Chem, catalog 81501) was assayed using a standard ELISA kit. All assays were performed according to the manufacturer’s instructions.

### Immunohistochemistry.

The small intestines (duodenum, jejunum, and ileum) from sham and VSG control mice were paraffin-embedded and sectioned onto slides by the University of Michigan In-Vivo Animal Core. Paraffin was removed using Citrisolv (VWR). The slides were incubated overnight at 4°C with primary antibody (see above). The slides were stained with corresponding secondary antibodies conjugated to fluorescence dyes (see above) for 2 hours at room temperature. The slides were mounted in an antifade fluorescence mounting medium containing DAPI (VECTASHIELD with DAPI, Vector Laboratories, catalog H-1200). Fluorescence images were obtained using an Olympus IX73 inverted fluorescence microscopy system or Nikon Eclipse Ti fluorescence microscope with a motorized X-Y stage system. Images were analyzed using Olympus cellSens imaging software or Image Pro 10 software (Media Cybernetics).

### FACS.

Four weeks after surgery, a liquid mixed meal was orally administered to overnight-fasted Lgr5^EGFP^ mice. At 15 minutes later, mice were euthanized with CO_2_ gas. The chest cavity was immediately opened, and saline was injected into the left ventricle to perfuse tissues. The small intestine was collected and flushed with cold PBS to remove luminal contents. Each 6 cm region (duodenum, jejunum, and ileum) was opened and placed into individual tubes containing cold DMEM (high glucose, 4.5 g/L) before immediately being processed for cell dissociation (STEMCELL Technologies, catalog 07174). Dissociated intestinal epithelial cells were stained with antibodies against CD31, CD45, and annexin V, and the Sytox Blue, and these cells were presorted with MoFlo Astrios cell sorter (Beckman Coulter) to rule out immune, endothelial, apoptotic, or dead cells, respectively. The cells were sorted into Norgen lysis buffer (Norgen Biotek 37500) on ice. The cells were vortexed and flash-frozen and stored at –80°C until being used. Intestinal epithelial cells from the WT mice were isolated and stained with APC, Sytox, and GFP for control.

### RNA library preparation and sequencing.

Total RNA was isolated from Lgr5^+^ cells, which were sorted by GFP expression, using the Total RNA Purification Kit (Norgen Biotek). RNA was quantified with the NanoDrop 2000 (Thermo Fisher Scientific), and RNA integrity was assessed by the Agilent 4200 Tapestation (Agilent Technologies). Libraries were prepared using NEBNext Ultra II Directional Library Prep Kit (New England Biolabs). Sequencing was performed on the NextSeq500 platform (Illumina) at the Biotechnology Research Center at Cornell University.

### RNA-Seq analysis.

The sequencing reads were aligned to the mouse genome (mm10). RNA-Seq reads were mapped to genome release using STAR (v2.5.3a) (48), and transcript quantification was performed using Salmon (v0.6.0) (49). Normalization and differential expression analysis were performed using DESeq2 (50). The Benjamini-Hochberg method was used for multiple-testing corrections. To define genes altered by VSG versus sham, samples from each of the regional segments (duodenum, jejunum, and ileum) were analyzed separately. One of the 3 jejunal samples with sham surgery and 1 of the 3 duodenal samples with sham surgery were excluded from the differential expression analysis due to low mapping percentages (below 25%). Two of the 4 VSG ileal samples and 1 of the 3 sham ileal samples were excluded due to low mapping percentages (below 15%). Raw sequencing data and normalized count files are available through Gene Expression Omnibus accession GSE173465.

### Mouse enteroid culture.

Male or female 12- to 20-week-old C57BL/6J or Gcg^Tomato^ mice were euthanized using CO_2_ gas. Duodenal or jejunal segments (~6 cm) were isolated and placed into Falcon tubes (Thermo Fisher Scientific) containing cold DMEM (high glucose, 4.5 g/L) containing Primocin (Invivogen, catalog ant-pm-1). Intestinal epithelial cells were dissociated using a Gentle Cell Dissociation Reagent (STEMCELL Technologies) and were filtered through a 70 μm cell strainer to remove villi. The remaining crypts were resuspended in Matrigel (growth factor reduced) and seeded into the prewarmed 24-well plate (1000 crypts/20 μL Matrigel). The enteroids were cultured using the IntestiCult Organoid Growth Medium (STEMCELL Technologies) for mice and placed in a CO_2_ incubator maintaining 5% CO_2_ concentration and 37°C temperature. The medium was changed every 2–3 days, and the enteroids were passaged every 7 days.

At least 7 days after the initial enteroid culture, enteroids were seeded into a 96-well, clear-bottom, black plate (10–20 enteroids/4–5 μL Matrigel/well). After 12 hours of acclimation time, we administered enteroids with various BAs or FXR agonists. Each reagent was prepared in DMSO and was administered to mouse duodenal or jejunal enteroids at each dose. For the enteroids derived from the Gcg^Tomato^ mice, we treated 4-hydroxytamoxifen (0.2 μg/mL) along with BAs. By doing so, the tamoxifen-dependent Cre driver was activated and we could visualize Gcg^+^ cells. The enteroids were imaged using an Olympus IX73 inverted fluorescence microscope system or a Biotek Cytation 5 fluorescence microscope system, and the images were analyzed using the Olympus cellSense or Gen5 Image Analysis software (Biotek). All results are representative results from 3 to 5 individual experiments.

For gene expression analysis, duodenum- or jejunum-derived mouse enteroids were seeded in 24-well dishes (4000 enteroids/80 μM Matrigel) and maintained for 12 hours for acclimation. Then, the enteroids were treated with vehicle or CA (20 μM) for 24 hours in a 37°C CO_2_ incubator. The results are representative results from 2 individual experiments performed in triplicate.

### Statistics.

GraphPad Prism 8.0 (GraphPad Software) was used for the statistical analysis. Data were considered significant when *P* < 0.05. Where applicable, a 2-tailed *t* test, an ordinary 2-way ANOVA, and a repeated measures ANOVA were applied to determine significant main effects and interactions between variables. Significant interactions are indicated in the figure legends, and between-group differences were determined by Tukey’s post hoc testing. A simple linear regression model was used to compare the plasma gut peptide levels by the corresponding EEC number. Data are presented as mean ± SEM. All the *P*adj and *P* values presented in the RNA-Seq analysis were determined by the Wald test (DESeq2). *P* < 0.05 was considered statistically significant.

### Study approval.

All animal experiments were performed according to an approved protocol by the Institutional Animal Care and Use Committee at the University of Michigan, and we followed protocols outlined in the NIH *Guide for the Care and Use of Laboratory Animals* (NIH Publication No. 8023, revised 1978).

## Author contributions

KSK and DAS conceptualized the study and were responsible for the design of experiments. KSK, BCEP, YHH, KKL, PHD, and LW were responsible for executing the experiments. KSK, RJS, JRS, PS, and DAS were responsible for analysis and interpretation of data. KSK and DAS were responsible for drafting of the manuscript. DAS provided final approval of the submitted manuscript.

## Supplementary Material

Supplemental data

## Figures and Tables

**Table 2:**
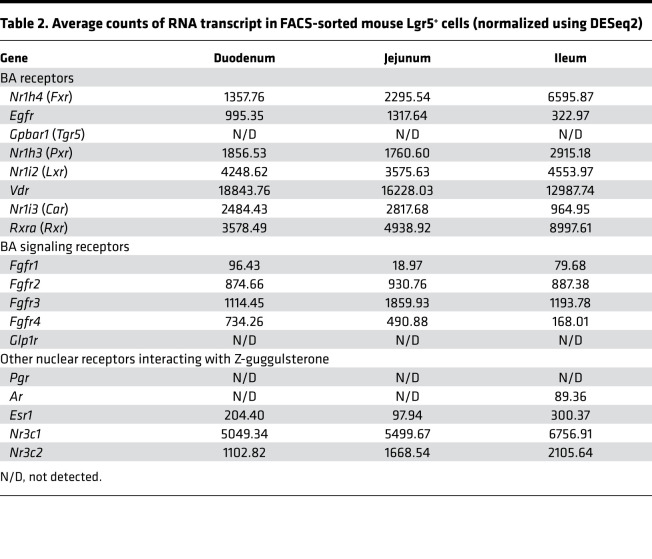
Average counts of RNA transcript in FACS-sorted mouse Lgr5^+^ cells (normalized using DESeq2)

**Table 1:**
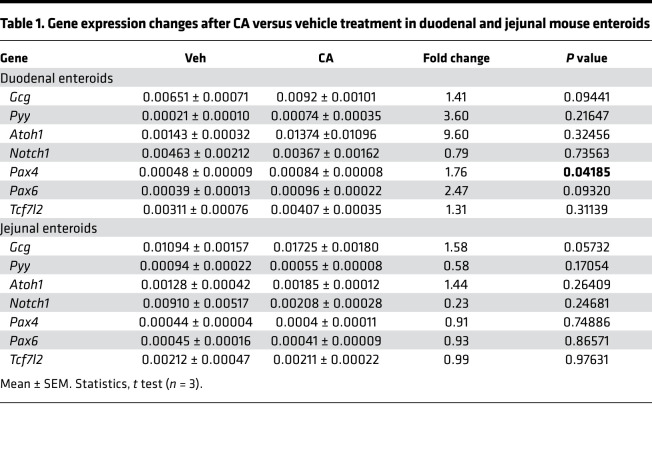
Gene expression changes after CA versus vehicle treatment in duodenal and jejunal mouse enteroids

## Version 1 05/03/2022

In-Press Preview

## Version 2 06/08/2022

Electronic publication
